# Blood pressure is associated with knee pain severity in middle-aged and elderly individuals with or at risks for osteoarthritis: data from the Osteoarthritis Initiative

**DOI:** 10.1186/s12891-024-07657-x

**Published:** 2024-07-12

**Authors:** Yao Liu, Guiying Du

**Affiliations:** 1 Department of Radiology, The Second Xiangya Hospital, Central South University, 139 Renmin Road, Furong District, Changsha, Hunan 410011 China; 2 Department of Radiology, Teda International Cardiovascular Hospital, Tianjin, China

**Keywords:** Blood pressure, Knee osteoarthritis, Pain, Hypertension

## Abstract

**Background:**

Hypertension is a common comorbidity of osteoarthritis (OA). Joint pain is the main clinical manifestation of OA. Knowledge about the relationship between hypertension and OA pain is limited. This study aimed to investigate whether blood pressure parameters are associated with knee pain severity in individuals with or at risks for OA.

**Methods:**

Our sample consisted of 2598 subjects (60.7% female, aged 45–79 years) collected from the Osteoarthritis Initiative. Blood pressure parameters included blood pressure stage, systolic blood pressure (SBP), diastolic blood pressure (DBP) and pulse pressure (PP). Radiographic evaluation using Kellgren-Lawrence system and pain severity evaluation using Western Ontario and McMaster Universities Osteoarthritis Index (WOMAC), Knee Injury and Osteoarthritis Outcome Score (KOOS), and Numeric Rating Scale (NRS) were performed for right knee. Linear regression was used to examine the relationship between blood pressure parameters and knee pain severity.

**Results:**

For the overall sample, blood pressure stage, SBP, and PP were positively correlated with WOMAC and NRS pain scores when adjusting for age, sex, and body mass index (BMI) (*p* ≤ 0.024) and were inversely correlated with KOOS score (*p* ≤ 0.004). After further adjusting for all covariates, PP remained a positive correlation with WOMAC score (*p* = 0.037) while other associations between blood pressure parameters and pain scores did not reach the statistical significance. In female, higher blood pressure stage, SBP, and PP were significantly associated with increased WOMAC and NRS scores and decreased KOOS score after adjustments of age and BMI (*p* ≤ 0.018). When adjusting for all covariates, the correlations of PP with WOMAC, KOOS and NRS scores remained significant (*p* = 0.008–0.049). In male sample, SBP was positively correlated with WOMAC score when adjusting for age and BMI (*p* = 0.050), but other associations between blood pressure parameters and pain scores were not statistically significant. No significant correlation was observed in male when further adjusting for other covariates.

**Conclusions:**

Increased PP is a risk factor for knee pain and mainly affects females, which suggested that controlling PP may be beneficial in preventing or reducing knee pain in females with or at risks for OA.

## Background

Osteoarthritis (OA) is one of the most common chronic arthritis worldwide, accounting for a significant proportion of the musculoskeletal disease burden [1, 2]. Recent investigations suggested that 7.6% of the world’s population (about 595 million individuals) lived with OA in 2020 [3]. Nonsurgical treatment strategies of OA include nonpharmacologic treatments (such as education, exercise, and weight loss if needed), anti-inflammatory agents (such as topical or oral non-steroidal anti-inflammatory drugs [NSAIDs]), intra-articular injections (typically corticosteroids and hyaluronic acid compounds), and additional pain medications (such as duloxetine, opioids). If non-operative treatment does not achieve satisfactory efficacy, then surgical treatments (arthroscopy, or total joint replacement) may be considered [1]. Joint replacement is restricted to end-stage OA patients with severely affected functional status, while the usage of total knee arthroplasty (TKA) and total hip arthroplasty (THA) was estimated to have a great increase in the coming decades, indicating an increasing disease burden [4].

Joint pain is the main clinical manifestation of OA, and it is the leading cause of mobility impairment in older adults, greatly influencing clinical decision-making [1]. Exploring the mechanism of OA pain is essential as a better understanding of the causes and risk factors of OA pain would be helpful in evaluation and management of OA. Although previous studies have suggested that joint structural abnormalities, inflammatory condition, neurobiological abnormalities, and psychosocial stress were associated with the incidence and severity of OA pain. However, despite these findings, the complex mechanism underlying OA pain remains poorly illustrated [5, 6].

Metabolic syndrome (MetS) is a common comorbidity of OA, which refers to a cluster of hypertension, dyslipidemia, abdominal obesity, and insulin resistance [7, 8]. In recent years, researchers have become increasingly concerned with the correlation between MetS and OA. By evaluating OA-related structural abnormalities and its changes over time, many studies have demonstrated that hypertension has a close relationship with the incidence and development of OA [7–14]. However, only a small number of studies investigated the association of hypertension with OA pain. A cross-sectional study by Shi et al. revealed a positive correlation between hypertension stages and knee pain severity, which was consistent with Li’s study [15, 16]. Nevertheless, Pan et al. reported an inverse correlation between blood pressure and “Mild pain” trajectory that defined as a mild level of pain consistent throughout the follow-up [17].

Among the studies mentioned, only the presence or stages of hypertension were included in analyses, whether more detailed blood pressure parameters (such as systolic blood pressure [SBP] and diastolic blood pressure [DBP]) are also associated with OA pain severity remained unknown. As little is known about the influence of hypertension on OA pain, further investigation needed. Clinically, a better understanding of the relationship between hypertension and OA pain would be important in identifying the risks of OA-related pain, as well as in developing corresponding countermeasures.

The purpose of this study was therefore to investigate whether and how blood pressure parameters are associated with knee pain severity in middle-aged and elderly patients with or at risks for OA.

## Materials and methods

### Database and subjects

This study utilized data from the Osteoarthritis Initiative (OAI), a longitudinal multicenter cohort study which recruited 4796 subjects with or at risk for knee OA. The OAI is sponsored by the US National Institutes of Health (NIH) and aiming to establish a public database to investigate the natural progression, risk factors, and predictors of knee OA. The OAI was approved by the institutional review board (IRB) of the OAI Coordinating Center at the University of California, San Francisco, and the IRBs of all participating centers. Written informed consent was obtained from all participants. The description of the OAI database is available at https://nda.nih.gov/oai/about-oai.

**Fig. 1:**
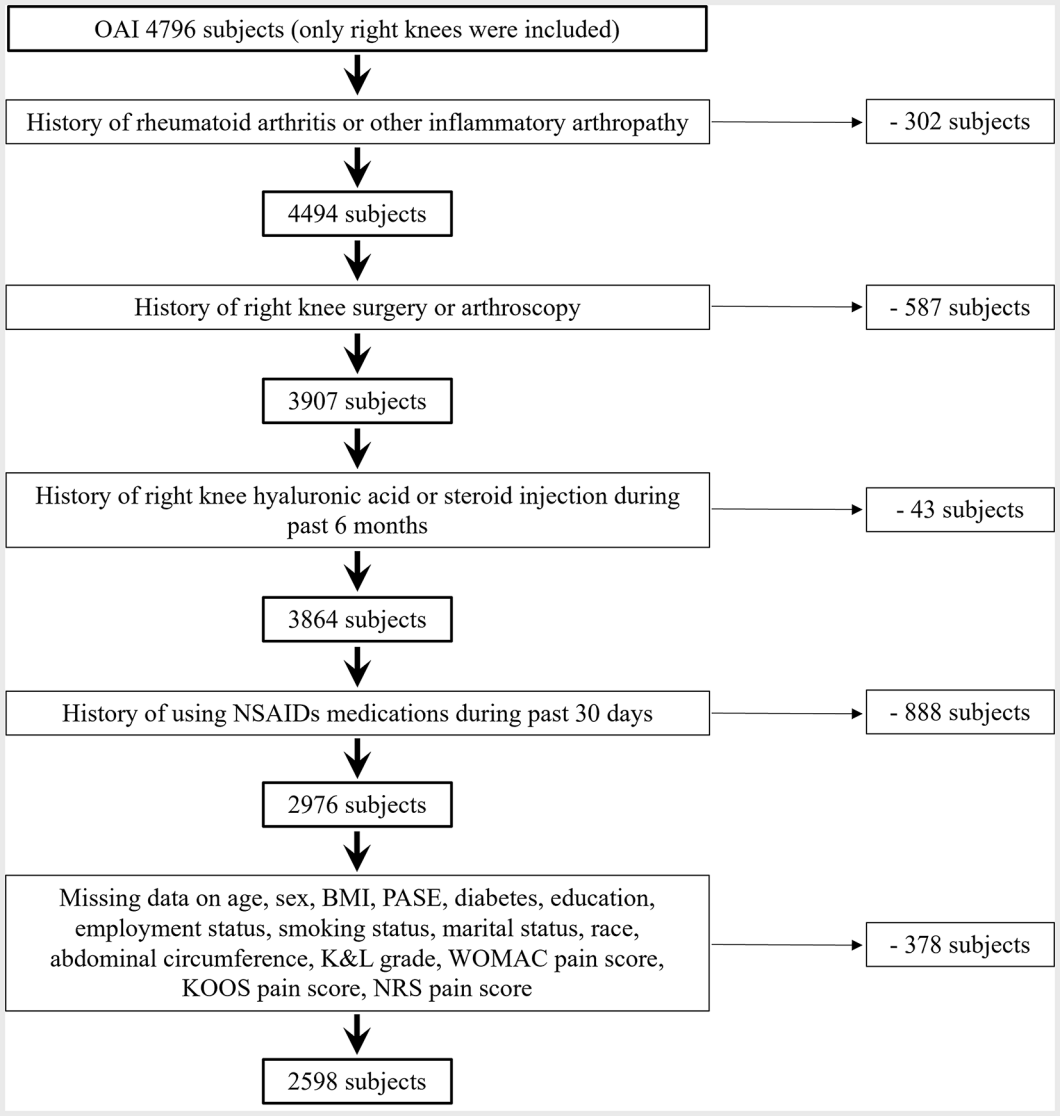
Flowchart demonstrating the process of sample selection. OAI, the osteoarthritis initiative. NSAIDs: non-steroidal anti-inflammatory drugs. BMI: body mass index. PASE: Physical Activity Score for the Elderly. K&L: Kellgren-Lawrence. WOMAC: Western Ontario and McMaster Universities Osteoarthritis Index. KOOS: Knee Injury and Osteoarthritis Outcome Score. NRS: Numeric Rating Scale

The study used only the right knee for assessment among the 4796 subjects, and applied the following exclusion criteria: (1) a history of rheumatoid arthritis or other inflammatory arthropathy; (2) a history of right knee surgery or arthroscopy; (3) a history of right knee hyaluronic acid or steroid injection during the past 6 months; (4) a history of using NSAIDs during the past 30 days; and (5) missing data on blood pressure measurements, knee pain assessments, medications and other covariates. The sample selection process is described in Fig. 1.

### Blood pressure

Blood pressure data were obtained from the OAI database (AllClinical_SAS, versions 0.2.3). Blood pressure measurements in the OAI study were performed by trained OAI staff using the auscultatory method. Participants were instructed to not drink any caffeine (from coffee, tea, or soda) and not eat or do any heavy physical activity, smoke, or ingest alcohol for 30 min prior to the blood pressure measurement. In addition, participants were asked to rest for approximately 5 min with their feet flat on the floor and legs and ankles uncrossed before measurement. Then, SBP and DBP were measured using a conventional mercury sphygmomanometer and stethoscope.

Based on the Seventh Report of the Joint National Committee on Prevention, Detection, Evaluation, and Treatment of High Blood Pressure (JNC 7), blood pressure in the present study was graded as follow: 0 = normal (SBP < 120 mm Hg and DBP < 80 mmHg); 1 = prehypertension (120 ≤ SBP ≤ 139 mm Hg or 80 ≤ DBP ≤ 89 mm Hg); 2 = stage 1 hypertension (140 ≤ SBP ≤ 159 mm Hg or 90 ≤ DBP ≤ 99 mm Hg); and 3 = stage 2 hypertension (SBP ≥ 160 mm Hg or DBP ≥ 100 mm Hg) [18]. In addition, pulse pressure (PP) was calculated by subtracting DBP from SBP.

### Knee pain severity

Data for knee pain assessment was obtained from the OAI database (AllClinical_SAS, versions 0.2.3). In the present study, Knee pain severity was evaluated using three different pain score systems, namely the Western Ontario and McMaster Universities Osteoarthritis Index (WOMAC), the Knee Injury and Osteoarthritis Outcome Score (KOOS), and the Numeric Rating Scale (NRS) for knee pain. The WOMAC pain score system includes 5 items associated with knee pain during different activities of daily life (i.e., walking, using stairs, in bed, sitting or lying, and standing) over the prior 7 days [19]. Each item is scored on a 0–4 Likert scale, with 0 indicating no pain and 4 indicating extreme pain. To represent knee pain severity, a sum score of the five items was calculated, with a possible sum score ranging from 0 to 20. The KOOS score is also used in evaluating knee pain for the past 7 days, which consisted of 9 items on a 0–4 Likert scale including twisting, straightening, bending knees, walking, going up or down stairs, sleeping, sitting or lying, and standing [20]. A normalized KOOS pain score (100 indicating no pain and 0 indicating extreme pain) was calculated. Besides, knee pain for the past 30 days was measured using NRS pain score [16]. Participants were asked “Please rate the pain that you’ve had in your right knee during the past 30 days by pointing to the number on this card that best describes the pain at its worst, with ‘0’ means ‘No pain’ and ‘10’ means ‘Pain as bad as you can imagine.’”

### Antihypertensive medications

The medication inventory data were obtained from the OAI database (MIF_SAS, versions 0.2.2). Participants were instructed to bring all prescription medications used in the preceding 30 days with them to the clinic visit. If a participant forgets to bring in one or more medications that they have taken in the last 30 days, the participant will be called 1 to 2 days after the visit to obtain the missing information. As previously described, the present study investigated commonly used prescription antihypertensive drugs, including beta-blockers, angiotensin converting enzyme inhibitor (ACEi), calcium channel blockers (CCBs), angiotensin receptor blocker (ARB) and thiazide diuretics [21].

### Other covariates

Demographic covariates contained age, sex (male or female), race (White/Caucasian or others [Black or African American, Asian, or other Non-white]), education (equal to/more than a college degree or less than a college degree), marital status (married or others [divorced, widowed, separated or never married], and employment status (works for pay or others [unpaid work or not working]). Clinical covariates included body mass index (BMI), Physical Activity Score for the Elderly (PASE), Kellgren-Lawrence (K&L) grade, smoking status (current smoker, or former smoker, or nonsmoker), diabetes, and abdominal circumference (measured in centimeter).

### Statistical analysis

Statistical analyses were performed using SPSS version.27 software (Chicago, IL), with a two-sided, 0.05 level of significance. For the characteristics of the study sample, summary statistics were constructed using frequencies and proportions for categorical data and means and standard deviations (SDs) for continuous variables. Linear regression analysis was performed to investigate the association between blood pressure parameters and knee pain severity scores.

## Results

Our sample consisted of 2598 subjects (60.7% female, mean age 61.7 ± 9.2 years [range 45–79 years], BMI 28.3 ± 4.7 kg/m^2^). The demographic and clinical characteristics of the sample were summarized in Table 1. Among the knees examined, 42.3% had K&L 0 (1100 knees), 19.4% had K&L 1 (503 knees), 26.8% had K&L 2 (697 knees), 10.0% had K&L 3 (260 knees), and 1.5% had K&L 4 (38 knees). In terms of blood pressure, 34.3% of the subjects had normal blood pressure (891 subjects), 46.3% had prehypertension (1204 subjects), 16.2% had stage 1 hypertension (421 subjects), and 3.2% had stage 2 hypertension (82 subjects).

**Table 1:** Characteristics of the study sample

Characteristics	*n* = 2598
Age, mean ± SD (years)	61.7 ± 9.2
Female [n (%)]	1576 (60.7%)
White/Caucasian [n (%)]	2128 (81.9%)
Body mass index, mean ± SD (kg/m^2^)	28.3 ± 4.7
PASE, mean ± SD	157.3 ± 78.4
Abdominal circumference, mean ± SD (cm)	101.8 ± 12.6
Diabetes [n (%)]	171 (6.6%)
Equal to or more than a college degree [n (%)]	1628 (62.7%)
Currently works for pay [n (%)]	1584 (61.0%)
Current smoker [n (%)]	68 (2.6%)
Married [n (%)]	1758 (67.7%)
Subjects on antihypertensive medications [n (%)]	1034 (39.8%)
Right knee K&L grades	
Grade 0 [n (%)]	1100 (42.3%)
Grade 1 [n (%)]	503 (19.4%)
Grade 2 [n (%)]	697 (26.8%)
Grade 3 [n (%)]	260 (10.0%)
Grade 4 [n (%)]	38 (1.5%)
Blood pressure stages	
Normal [n (%)]	891 (34.3%)
Prehypertension [n (%)]	1204 (46.3%)
Stage 1 hypertension [n (%)]	421 (16.2%)
Stage 2 hypertension [n (%)]	82 (3.2%)
WOMAC pain scores, mean ± SD	1.8 ± 2.7
KOOS pain scores, mean ± SD	87.8 ± 14.6
NRS pain scores, mean ± SD	2.2 ± 2.5

SD: standard deviation. PASE: Physical Activity Score for the Elderly. K&L: Kellgren-Lawrence. WOMAC: Western Ontario and McMaster Universities Osteoarthritis Index. KOOS: Knee Injury and Osteoarthritis Outcome Score. NRS: Numeric Rating Scale

As shown in Table 2, after adjusting for age, sex, and BMI (Model 1), higher blood pressure stage was statistically correlated with higher WOMAC pain score (β = 0.21, 95% CI [Confidence interval]: 0.08 to 0.35, *p* = 0.002) and NRS pain score (β = 0.15, 95% CI: 0.02 to 0.28, *p* = 0.020), and increased blood pressure stage was significantly associated with decreased KOOS pain score (β = -1.08, 95% CI: -1.81 to -0.34, *p* = 0.004). Similarly, SBP and PP were positively associated with WOMAC pain score (β = 0.01, 95% CI: 0.01 to 0.02, *p* < 0.001 for SBP; β = 0.01, 95% CI: 0.01 to 0.02, *p* < 0.001 for PP) and NRS pain score (β = 0.01, 95% CI: 0.00 to 0.01, *p* = 0.024 for SBP; β = 0.01, 95% CI: 0.00 to 0.02, *p* = 0.004 for PP) and were also inversely associated with KOOS pain score (β = -0.06, 95% CI: -0.10 to -0.02, *p* = 0.002 for SBP; β = -0.07, 95% CI: -0.12 to -0.03, *p* = 0.003 for PP), which were statistically significant. However, DBP did not show a statistical association with knee pain scores. In model 2, after further adjusting for some covariates (including PASE, diabetes, education, employment status, smoking, marital status, race, abdominal circumference), higher PP had a significant correlation with higher WOMAC pain score (β = 0.01, 95% CI: 0.00 to 0.02, *p* = 0.034), but the association of PP with KOOS and NRS pain scores did not reach the statistical significance. The correlation between blood pressure stage, SBP and DBP and pain scores was not significant. When further adjusting for K&L grade (Model 3), higher PP was significantly associated with higher WOMAC pain score (β = 0.01, 95% CI: 0.00 to 0.02, *p* = 0.026) and NRS pain score (β = 0.01, 95% CI: 0.00 to 0.02, *p* = 0.047) and had a statistically association with decreased KOOS pain score (β = -0.05, 95% CI: -0.09 to -0.00, *p* = 0.043). Blood pressure stage, SBP and DBP did not show a significant correlation with pian scores in this model. In model 4 (further adjusted for antihypertensive medications), only a significant association between PP and WOMAC pian score (β = 0.01, 95% CI: 0.00 to 0.02, *p* = 0.037) was observed, while blood pressure stage, SBP and DBP did not demonstrate a significant correlation with pain scores.

**Table 2:** Association between blood pressure parameters and knee pain severity scores for the overall sample

Parameters		WOMAC score			KOOS score			NRS score	
		β (95% CI)	*p* value		β (95% CI)	*p* value		β (95% CI)	*p* value
**Model 1**									
Blood pressure stage		0.21 (0.08, 0.35)	**0.002**		-1.08 (-1.81, -0.34)	**0.004**		0.15 (0.02, 0.28)	**0.020**
SBP		0.01 (0.01, 0.02)	**< 0.001**		-0.06 (-0.10, -0.02)	**0.002**		0.01 (0.00, 0.01)	**0.024**
DBP		0.01 (-0.0.01, 0.02)	0.309		-0.03 (-0.09, 0.03)	0.301		0.00 (-0.01, 0.01)	0.952
PP		0.01 (0.01, 0.02)	**< 0.001**		-0.07 (-0.12, -0.03)	**0.003**		0.01 (0.00, 0.02)	**0.004**
**Model 2**									
Blood pressure stage		0.11 (-0.02, 0.24)	0.096		-0.56 (-1.28, 0.17)	0.133		0.0 (-0.06, 0.19)	0.295
SBP		0.01 (-0.00, 0.01)	0.111		-0.03 (-0.06, 0.01)	0.140		0.00 (-0.00, 0.01)	0.430
DBP		-0.00 (-0.01, 0.01)	0.829		0.00 (-0.06, 0.06)	0.917		-0.01 (-0.02, 0.00)	0.238
PP		0.01 (0.00, 0.02)	**0.034**		-0.04 (-0.09, 0.00)	0.059		0.01 (-0.00, 0.02)	0.062
**Model 3**									
Blood pressure stage		0.10 (-0.03, 0.23)	0.141		-0.46 (-1.17, 0.25)	0.205		0.05 (-0.07, 0.18)	0.411
SBP		0.01 (-0.00, 0.01)	0.147		-0.02 (-0.06, 0.01)	0.194		0.00 (-0.00, 0.01)	0.540
DBP		-0.00 (-0.01, 0.01)	0.553		0.02 (-0.04, 0.08)	0.565		-0.01 (-0.02, 0.00)	0.103
PP		0.01 (0.00, 0.02)	**0.026**		-0.05 (-0.09, -0.00)	**0.043**		0.01 (0.00, 0.02)	**0.047**
**Model 4**									
Blood pressure stage		0.09 (-0.04, 0.22)	0.163		-0.43 (-1.15, 0.28)	0.232		0.05 (-0.07, 0.17)	0.433
SBP		0.00 (-0.00, 0.01)	0.183		-0.02 (-0.06, 0.01)	0.238		0.00 (-0.01, 0.01)	0.589
DBP		-0.00 (-0.01, 0.01)	0.563		0.02 (-0.04, 0.08)	0.582		-0.01 (-0.02, 0.00)	0.105
PP		0.01 (0.00, 0.02)	**0.037**		-0.04 (-0.09, 0.00)	0.061		0.01 (-0.00, 0.02)	0.057

β is the regression coefficient. P value is for the regression coefficient, and boldface P value shows statistically significant result (*p* < 0.05). CI: Confidence interval. WOMAC: Western Ontario and McMaster Universities Osteoarthritis Index. KOOS: Knee Injury and Osteoarthritis Outcome Score. NRS: Numeric Rating Scale. SBP: systolic blood pressure. DBP: diastolic blood pressure. PP: pulse pressure

Model 1: adjusted for age, sex, and BMI

Model 2: adjusted for Model 1and PASE, diabetes, education, employment status, smoking, marital status, race, abdominal circumference

Model 3: adjusted for Model 2 + K&L grade

Model 4: adjusted for Model 3 + usage of each type of antihypertensive medications

Table 3 showed the association between blood pressure parameters and knee pain severity scores in female. In model 1, blood pressure stage, SBP and PP were positively correlated with WOMAC pain score (β = 0.27, 95% CI: 0.08 to 0.45, *p* = 0.005 for blood pressure stage; β = 0.01, 95% CI: 0.00 to 0.02, *p* = 0.007 for SBP; β = 0.02, 95% CI: 0.01 to 0.03, *p* = 0.003 for PP) and NRS pain score (β = 0.21, 95% CI: 0.04 to 0.38, *p* = 0.018 for blood pressure stage; β = 0.01, 95% CI: 0.00 to 0.02, *p* = 0.007 for SBP; β = 0.02, 95% CI: 0.01 to 0.03, *p* < 0.001 for PP), and were inversely correlated with KOOS pain score (β = -1.34, 95% CI: -2.34 to -0.33, *p* = 0.009 for blood pressure stage; β = -0.06, 95% CI: -0.11 to -0.01, *p* = 0.012 for SBP; β = -0.09, 95% CI: -0.15 to -0.03, *p* = 0.006 for PP). DBP did not show a statistical association with pain scores in this model. In model 2, higher PP had a significant relation with increased WOMAC pain score (β = 0.01, 95% CI: 0.00 to 0.02, *p* = 0.036) and NRS (β = 0.01, 95% CI: 0.00 to 0.03, *p* = 0.009), while blood pressure stage, SBP and DBP did not show a statistical association with pain scores. After further adjusted for K&L grade (model 3), PP was significantly correlated with WOMAC, KOOS and NRS scores (β = 0.01, 95% CI: 0.00 to 0.02, *p* = 0.028 for WOMAC; β = -0.06, 95% CI: -0.12 to -0.00, *p* = 0.038 for KOOS; β = 0.02, 95% CI: 0.00 to 0.03, *p* = 0.006 for NRS), but blood pressure stage, SBP and DBP were not significantly correlated with pain scores. Similar results were observed in model 4 (further adjusted for antihypertensive medications) as in model 3.

**Table 3:** Association between blood pressure parameters and knee pain severity scores in female

Parameters		WOMAC score			KOOS score			NRS score	
		β (95% CI)	*p* value		β (95% CI)	*p* value		β (95% CI)	*p* value
**Model 1**									
Blood pressure stage		0.27 (0.08, 0.45)	**0.005**		-1.34 (-2.34, -0.33)	**0.009**		0.21 (0.04, 0.38)	**0.018**
SBP		0.01 (0.00, 0.02)	**0.007**		-0.06 (-0.11, -0.01)	**0.012**		0.01 (0.00, 0.02)	**0.007**
DBP		0.00 (-0.01, 0.02)	0.602		-0.02 (-0.10, 0.06)	0.627		0.00 (-0.01, 0.01)	0.981
PP		0.02 (0.01, 0.03)	**0.003**		-0.09 (-0.15, -0.03)	**0.006**		0.02 (0.01, 0.03)	**< 0.001**
**Model 2**									
Blood pressure stage		0.14 (-0.04, 0.33)	0.130		-0.66 (-1.66, 0.33)	0.192		0.11 (-0.06, 0.28)	0.218
SBP		0.01 (-0.00, 0.02)	0.260		-0.03 (-0.08, 0.03)	0.324		0.01 (-0.00, 0.02)	0.173
DBP		-0.01 (-0.02, 0.01)	0.359		0.04 (-0.04, 0.12)	0.350		-0.01 (-0.02, 0.01)	0.220
PP		0.01 (0.00, 0.02)	**0.036**		-0.06 (-0.12, 0.00)	0.053		0.01 (0.00, 0.03)	**0.009**
**Model 3**									
Blood pressure stage		0.13 (-0.05, 0.31)	0.163		-0.58 (-1.55, 0.40)	0.248		0.09 (-0.08, 0.26)	0.274
SBP		0.01 (-0.00, 0.01)	0.314		-0.02 (-0.07, 0.03)	0.406		0.01 (-0.00, 0.01)	0.219
DBP		-0.01 (-0.02, 0.01)	0.217		0.05 (-0.02, 0.13)	0.175		-0.01 (-0.03, 0.00)	0.108
PP		0.01 (0.00, 0.02)	**0.028**		-0.06 (-0.12, -0.00)	**0.038**		0.02 (0.00, 0.03)	**0.006**
**Model 4**									
Blood pressure stage		0.12 (-0.07, 0.30)	0.212		-0.51 (-1.50, 0.47)	0.305		0.09 (-0.08, 0.26)	0.310
SBP		0.00 (-0.01, 0.01)	0.389		-0.02 (-0.07, 0.03)	0.474		0.01 (-0.00, 0.01)	0.259
DBP		-0.01 (-0.02, 0.01)	0.196		0.06 (-0.02, 0.13)	0.162		-0.01 (-0.03, 0.00)	0.104
PP		0.01 (0.00, 0.02)	**0.039**		-0.06 (-0.12, -0.00)	**0.049**		0.01 (0.00, 0.03)	**0.008**

β is the regression coefficient. P value is for the regression coefficient, and boldface P value shows statistically significant result (*p* < 0.05). CI: Confidence interval. WOMAC: Western Ontario and McMaster Universities Osteoarthritis Index. KOOS: Knee Injury and Osteoarthritis Outcome Score. NRS: Numeric Rating Scale. SBP: systolic blood pressure. DBP: diastolic blood pressure. PP: pulse pressure

Model 1: adjusted for age and BMI

Model 2: adjusted for Model 1 + PASE, diabetes, education, employment status, smoking, marital status, race, abdominal circumference

Model 3: adjusted for Model 2 + K&L grade

Model 4: adjusted for Model 3 + usage of each type of antihypertensive medications

Table 4 showed that, in male, SBP was significantly associated with WOMAC pain score when adjusting for age and BMI (β = 0.01, 95% CI: 0.00 to 0.02, *p* = 0.050) but did not show a significant correlation with KOOS and NRS pain scores. Blood pressure stage, DBP and PP were not statistically associated with pain scores in this model. In addition, we did not observe a significant association between blood pressure parameters and pain scores in model 2, 3 and 4.

**Table 4:** Association between blood pressure parameters and knee pain severity scores in male

Parameters		WOMAC score			KOOS score			NRS score	
		β (95% CI)	*p* value		β (95% CI)	*p* value		β (95% CI)	*p* value
**Model 1**									
Blood pressure stage		0.14 (-0.04, 0.33)	0.132		-0.80 (-1.86, 0.27)	0.142		0.07 (-0.11, 0.26)	0.435
SBP		0.01 (0.00, 0.02)	**0.050**		-0.05 (-0.11, 0.00)	0.063		0.00 (-0.01, 0.01)	0.870
DBP		0.01 (-0.01, 0.02)	0.328		-0.05 (-0.14, 0.04)	0.272		-0.00 (-0.02, 0.01)	0.839
PP		0.01 (-0.00, 0.02)	0.096		-0.05 (-0.12, 0.02)	0.146		0.00 (-0.01, 0.01)	0.725
**Model 2**									
Blood pressure stage		0.07 (-0.12, 0.24)	0.481		-0.40 (-1.43, 0.63)	0.448		0.00 (-0.18, 0.18)	0.984
SBP		0.01 (-0.00, 0.01)	0.278		-0.03 (-0.08, 0.02)	0.276		-0.00 (-0.01, 0.01)	0.499
DBP		0.01 (-0.01, 0.02)	0.373		-0.05 (-0.14, 0.04)	0.268		-0.00 (-0.02, 0.01)	0.681
PP		0.00 (-0.01, 0.02)	0.506		-0.02 (-0.08, 0.05)	0.609		-0.00 (-0.01, 0.01)	0.603
**Model 3**									
Blood pressure stage		0.05 (-0.13, 0.23)	0.563		-0.32 (-1.33, 0.70)	0.539		-0.01 (-0.19, 0.17)	0.899
SBP		0.01 (-0.00, 0.01)	0.302		-0.03 (-0.08, 0.03)	0.302		-0.00 (-0.01, 0.01)	0.446
DBP		0.01 (-0.01, 0.02)	0.491		-0.04 (-0.13, 0.05)	0.378		-0.01 (-0.02, 0.01)	0.516
PP		0.00 (-0.01, 0.02)	0.454		-0.02 (-0.08, 0.04)	0.544		-0.00 (-0.01, 0.01)	0.652
**Model 4**									
Blood pressure stage		0.06 (-0.12, 0.24)	0.493		-0.36 (-1.38, 0.66)	0.485		-0.01 (-0.19, 0.17)	0.940
SBP		0.01 (-0.00, 0.01)	0.277		-0.03 (-0.08, 0.02)	0.280		-0.00 (-0.01, 0.01)	0.466
DBP		0.01 (-0.01, 0.02)	0.465		-0.04 (-0.13, 0.05)	0.368		-0.01 (-0.02, 0.01)	0.534
PP		0.01 (-0.01, 0.02)	0.431		-0.02 (-0.09, 0.04)	0.515		-0.00 (-0.01, 0.01)	0.668

β is the regression coefficient. P value is for the regression coefficient, and boldface P value shows statistically significant result (*p* < 0.05). CI: Confidence interval. WOMAC: Western Ontario and McMaster Universities Osteoarthritis Index. KOOS: Knee Injury and Osteoarthritis Outcome Score. NRS: Numeric Rating Scale. SBP: systolic blood pressure. DBP: diastolic blood pressure. PP: pulse pressure

Model 1: adjusted for age and BMI

Model 2: adjusted for Model 1 + PASE, diabetes, education, employment status, smoking, marital status, race, abdominal circumference

Model 3: adjusted for Model 2 + K&L grade

Model 4: adjusted for Model 3 + usage of each type of antihypertensive medications

## Discussion

In the present study, we aimed to explore the relationship between blood pressure and knee pain severity in individuals with or at risks for osteoarthritis. We found that, after adjustments, PP was positively associated with WOMAC pain score for the overall sample, and this correlation remained significant in female but did not reach the statistical significance in male. This finding indicated that the influence of blood pressure on knee joint pain severity mainly exists in female group. In addition, among the blood pressure parameters, PP is the key factor associating with knee pain severity.

Osteoarthritis is a whole joint disease, with a complex pathological process involves biomechanics, inflammation, metabolism, and other factors, and may affect various joint structures like cartilage, subchondral bone, ligaments, muscles, and menisci. As the main clinical symptom of OA, joint pain is modified by not only clinical factors but also external characteristics [1]. Previous research has shown that factors related to OA pain involve multiple aspects, including biological, psychological, behavioral, and sociological factors. These factors are involved in the generation, conduction, and perception of pain to varying degrees [1]. In early studies, it was thought that the degree of joint structural damage should be proportional to the degree of joint pain, but later studies have found that this is not always the case. With the expansion and deepening of research, various new factors related to joint pain are constantly being discovered, such as metabolic factors, sex, emotional factors, drugs, socioeconomic status [22–25].

In recent years, numerous studies have examined the relationship between MetS and OA and have found that hypertension is closely associated with the incidence and progression of OA [7–13]. However, only a few studies have investigated how hypertension correlates with OA pain. Li et al. and Alenazi et al. reported that the presence of hypertension was associated with joint pain severity [15, 26]. Using three different scoring instruments for knee pain evaluation, Shi et al. demonstrated that hypertension was associated with both short-term (past 48 h) and long-term (past 7 days and 30 days) knee pain severity among OA patients [16]. Although these cross-sectional studies provided evidence for an association between hypertension and OA pain, there are still some factors that require further consideration. For example, previous studies have reported that radiographic OA severity is closely related to knee pain severity, which was not included in the analysis in Li’s and Alenazi’s studies [27–29]. In the current study, we added K&L grade into regression models and found that PP was associated with knee pain severity independently of K&L grade. Additionally, recent studies have shown that certain types of antihypertensive medications also have an effect on knee pain severity, which has not been considered in previous studies mentioned above [21, 30]. For instance, among the commonly used antihypertensive medications, calcium channel blockers (CCBs) were found to be associated with high WOMAC pain score, as well as high knee replacement rates and less knee joint space width. These effects were speculated to arise from the influence of CCBs on articular chondrocyte activity and skeletal muscle function. Moreover, the antagonistic interaction between NSAIDs and CCBs on blood vessels could potentially compromise blood pressure regulation, thereby impacting the progression of knee OA. Furthermore, the use of beta-blockers was revealed to be associated with less joint pain and a lower use of opioids and other analgesics in patients with symptomatic large-joint OA. This could be attributed to the inhibitory effect of adrenergic downregulation on certain enzymes and cytokines associated with degenerative processes in the joints [31]. Overall, these findings underscore the complex interplay between hypertension, antihypertensive medications, and OA pain, warranting further investigation. In comparison with previous works, our study investigated a more extensive range of blood pressure parameters and covariates, which allowed this study to have a better investigation of the correlation of blood pressure on OA knee pain severity. The current study suggested that the association between blood pressure and knee pain severity was independent of K&L grade. More importantly, we found that PP may play a key role in the association between blood pressure and knee pain severity, as there was no significant association between SBP, DBP, and blood pressure stage and knee pain scores after adjustments. Furthermore, in the overall sample, although PP remained significantly associated with WOMAC pain score after adjustments, the significance of this association disappeared in male sample. However, the correlation between PP and each pain score remained significant in female group after adjustments. This finding suggested that the effect of blood pressure on knee pain severity may only exist in females and was independent of antihypertensive medication usage.

In a longitudinal study, Niu and colleagues reported an association between high blood pressure and incident symptomatic knee OA (defined as present when a knee developed both radiographic OA and knee pain), indicating that hypertension may be a predictor for the occurrence of knee pain [32]. However, as the outcome parameter was the combination of radiographic OA and knee pain, the independent effects of blood pressure on knee pain could not be identified. Another study by Pan co-workers investigated the influence of MetS on knee pain trajectories using group-based trajectory modelling, where they described three different pain trajectories: “minimal pain”, “mild pain”, and “moderate pain” [17]. They found an independent and inverse association between high blood pressure and the “mild pain” trajectory, which was defined as a mild level of pain consistent throughout the follow-up period of 10.7 years. These findings suggested that, longitudinally, there may be a potential complex interrelationship between hypertension and OA pain. However, to date, direct evidence of the relationship between hypertension and the occurrence and progression of knee pain is still lacking.

Although the underlying mechanism of association between hypertension and OA is not well elucidated so far, a theory of subchondral ischemia was proposed to explain the influence of hypertension on OA [33–36]. Hypertension-related abnormalities, such as elevated intraosseous pressure and rarefaction of subchondral micro vessels, may compromise subchondral blood perfusion and impair the nutrient and oxygen supply to the cartilage, leading to its degradation. This theory was supported by Aaron’s study that found a reduction of arterial inflow and delayed signal enhancement in subchondral area in OA knees using dynamic contrast-enhanced MRI [37]. Recent studies have also reported more severe knee cartilage abnormalities in individuals with higher blood pressure [9, 10]. At the cellular and molecular levels, hypertension and OA share some common molecular pathways, such as the renin-angiotensin system, endothelin system and Wnt-β-catenin signalling pathway [36]. These molecular pathways are interconnected and play an essential role in controlling vascular tone. In hypertension, these pathways are upregulated, and they may induce chondrocyte hypertrophy and an inflammatory response, leading to joint catabolism and ultimately contributing to the initiation and development of OA. During the process of OA development, infiltration of nerve endings and expression of inflammatory factors were observed in subchondral area, which were speculated to be involved in pain sensation generation [36]. However, knowledge about the exact mechanisms underlying the relationship between blood pressure and OA pain are limited, further research is needed to fully elucidate the mechanisms and to develop effective interventions for individuals with hypertension who are at risk of developing OA pain.

We acknowledge that our study has certain limitations that need to be considered. Firstly, due to the cross-sectional design, our study was unable to establish a causal relationship between blood pressure and OA pain severity, which could only be achieved through further longitudinal studies. Secondly, only limited variables were included in our analytical model, and given the complexity of joint pain mechanisms, more potentially relevant factors need to be further examined in future studies. However, despite these limitations, we believe this study is valuable and provides novel information on the correlation between blood pressure and OA pain.

## Conclusion

This study investigated the relationship between blood pressure and OA-related knee pain severity. We found a significant correlation between PP and each knee pain scores in female group, suggesting that increased PP is a risk factor for knee pain and mainly affects females. Our study provided new insights on relationship between blood pressure and knee pain and indicated that controlling PP may be beneficial in preventing or reducing knee pain in females with or at risks for OA.

## Data Availability

The datasets generated and/or analyzed during the current study are available from the Osteoarthritis Initiative (OAI; https://nda.nih.gov/oai).

## Abbreviations

OA: Osteoarthritis
MetS: Metabolic syndrome
SBP: Systolic blood pressure.
DBP: Diastolic blood pressure.
OAI: The Osteoarthritis Initiative
NIH: The US National Institutes of Health
IRB: The institutional review board
NSAIDs: Non-steroidal anti-inflammatory drugs
BMI: Body mass index
PASE: Physical Activity Score for the Elderly
K&L: Kellgren-Lawrence
WOMAC: Western Ontario and McMaster Universities Osteoarthritis Index
KOOS: Knee Injury and Osteoarthritis Outcome Score
NRS: Numeric Rating Scale
JNC 7: The Seventh Report of the Joint National Committee on Prevention, Detection, Evaluation, and Treatment of High Blood Pressure
PP: Pulse pressure
ACEi: Angiotensin converting enzyme inhibitor
CCBs: Calcium channel blockers
ARB: Angiotensin receptor blocker
SD: Standard deviation
TKA: Total knee arthroplasty
THA: Total hip arthroplasty
